# Recreating the village: the patient experience with a hybrid model of Group Perinatal Care (GPPC) in an academic family health team

**DOI:** 10.1186/s12884-024-06405-2

**Published:** 2024-04-02

**Authors:** Anne Biringer, Natalie Morson, Sakina Walji, Natalie Tregaskiss, Susannah Merritt, Tutsirai Makuwaza, Milena Forte

**Affiliations:** 1 Ray D. Wolfe Department of Family Medicine, Sinai Health, 60 Murray Street, Box 25, Toronto, ON M5T 3L9 Canada; 2 Department of Family and Community Medicine, University of Toronto, Toronto, ON Canada

**Keywords:** Perinatal, Group, Mental health, Pregnancy, Social support

## Abstract

**Background:**

Group prenatal care (GPC) has been shown to have a positive impact on social support, patient knowledge and preparedness for birth. We developed an interprofessional hybrid model of care whereby the group perinatal care (GPPC) component was co-facilitated by midwives (MW) and family medicine residents (FMR) and alternating individual visits were provided by family physicians (FP’s) within our academic family health team (FHT) In this qualitative study, we sought to explore the impact of this program and how it supports patients through pregnancy and the early newborn period.

**Methods:**

Qualitative study that was conducted using semi-structured telephone interviews with 18 participants who had completed GPPC in the Mount Sinai Academic Family Health Team in Toronto, Canada and delivered between November 2016 and October 2018. Interviews were audio-recorded and transcribed verbatim. Thematic analysis was conducted by team members using grounded theory**.**

**Results:**

Four over-arching themes emerged from the data: (i) Participants highly valued information they received from multiple trusted sources, (ii) Participants felt well cared for by the collaborative and coordinated interprofessional team, (iii) The design of GPPC enabled a shared experience, allowing for increased support of the pregnant person, and (iv) GPPC facilitated a supportive transition into the community which positively impacted participants’ emotional well- being.

**Conclusions:**

The four constructs of social support (emotional, informational, instrumental and appraisal) were central to the value that participants found in GPPC. This support from the team of healthcare providers, peers and partners had a positive impact on participants’ mental health and helped them face the challenges of their transition to parenthood.

## Introduction

Group prenatal care (GPC) offers a “one stop” approach to clinical care and perinatal education. While traditional prenatal care is separated into individual care with a health care practitioner and ancillary prenatal education, GPC is based on Centering Pregnancy [1], a model of group prenatal care developed in 1994 which includes risk assessment, education and support delivered in a group setting. The program is based on the premise that pregnant individuals should be equal partners in care. Group prenatal care has been shown to be associated with high levels of patient satisfaction and some improved clinical outcomes [2, 3]. Manant et al.’s integrative literature review identified individual and community level outcomes of this model [4]. They grouped individual outcomes into capacity building (ability to make informed decisions), psychological issues (depression and stress reduction), social connection (social support) and optimal pregnancy course (of complications and patient’s satisfaction). Other outcomes were measured at the community level. The authors pointed out some of the limitations in the quality of the research and a number of areas for future study. In addition, they suggested the need for more qualitative research to assess how participation in this model may affect participants. Despite these cautions, in 2016 the World Health Organization (WHO) recommended group antenatal care as a health systems intervention aimed at improving the utilization and quality of antenatal care - recognizing the importance of women’s experience of care [5].

Centering pregnancy has been extended to Centering Parenting in some settings [6]. In this model, parents play a pivotal role in group well child visits with health care providers who also facilitate group discussions for the parents. Again, positive results have been reported such as social interaction between parents and health care staff as well as increasing the number of well-baby visits, immunization rates and breastfeeding at 6 months [7]. However studies are limited in their generalizability and more high quality, longitudinal research is needed to elucidate long term population outcomes and cost effectiveness [8].

Qualitative research has tried to explore the patient experience further. It reveals high satisfaction with the group prenatal care model [9] for many reasons including: increased connections, learning from the group, preparedness for birth, normalization of the birth experience and improved relationships. Other GPC research highlights the importance of respect, the power of knowledge, factors enabling patients to be better mothers and the importance of mutual support among participants [10]. More recently, Renbarger described four constructs of social support provided by the group prenatal care structure:—informational (advice, suggestions and information to assist with problem solving), emotional (expressions of empathy, trust, caring), instrumental (tangible assistance) and appraisal (constructive feedback provided for assessment and encouragement) support [11]. They outlined how each promoted learning, prepared women for motherhood, improved emotional well-being and helped women build relationships with peers and health care providers.

GPC provided in the family medicine teaching setting has also been studied. GPC co-facilitated by a family medicine faculty member and a first year and second family medicine resident has shown improvements in both care processes and outcomes such as preterm birth and Cesarean Section [12]. In fact, residency programs with GPC models report more graduates entering OB fellowships and practicing maternity care [13]. However, the impact of resident-led programs on the patient experience has not been examined.

In recent years there has been much greater appreciation of the importance of mental health and social support in the perinatal period with numerous medical and national associations providing guidance on screening for and treating mental health conditions and psychosocial distress in this vulnerable time [14–16]. The significant incidence of perinatal anxiety and depression with their links to adverse maternal and childhood outcomes make it imperative to pay attention to this once neglected aspect of perinatal care [17–19]. Given the evidence for the impact of GPC on social support, patient knowledge and preparedness for birth, we developed a hybrid interprofessional model of group perinatal care (GPPC) and individual perinatal care within our academic family health team (FHT). We are deliberate in the use of the term group “perinatal” care as opposed to the traditional “prenatal” care since the group sessions continue well into the postpartum period and the medical care of parent and baby often continues with the family health team long past the traditional postpartum period.

In this qualitative study, we sought to explore the impact of this care and how this model supports patients through pregnancy and the early newborn period.

### Context

**Fig. 1:**
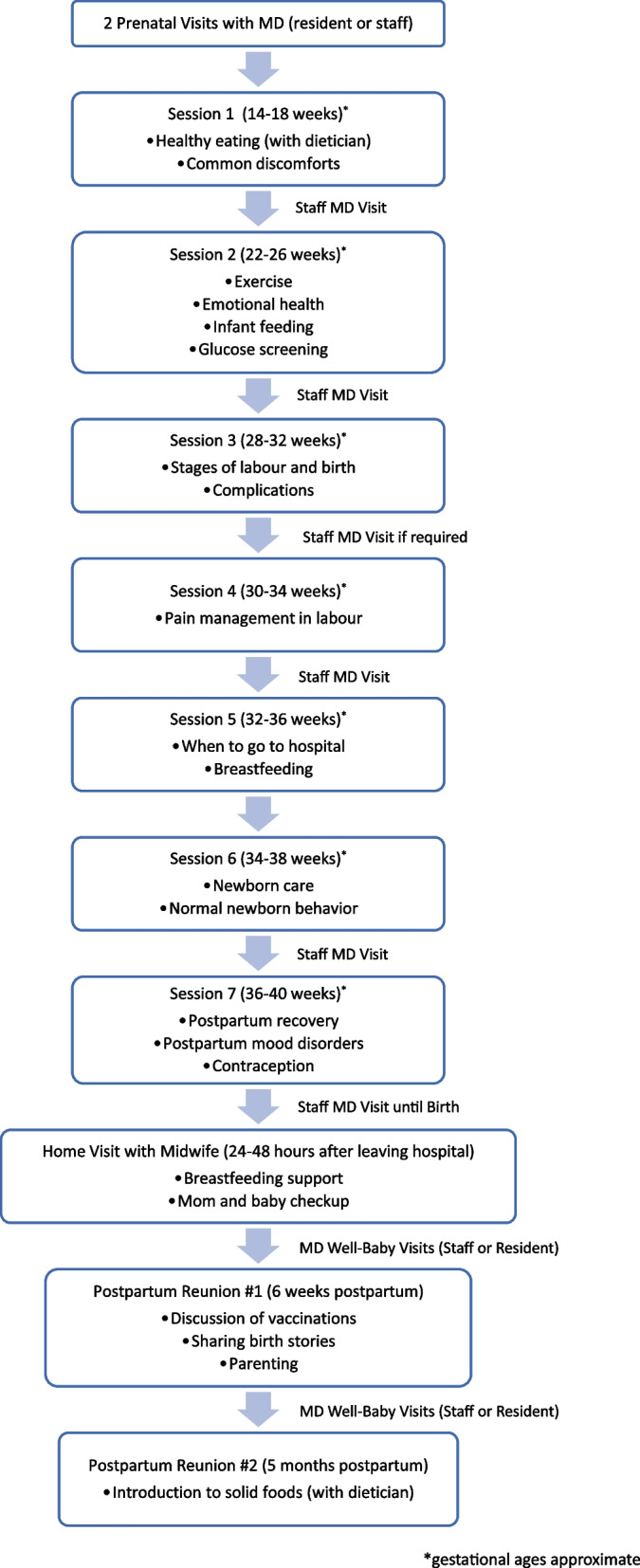
The group perinatal care pathway

The Mount Sinai Academic Family Health Team (MSAFHT), a division of the University of Toronto Department of Family and Community Medicine in Toronto, Canada, developed an interprofessional hybrid model of care whereby the group perinatal care (GPPC) component was co-facilitated by midwives (MW) and family medicine residents (FMR) and alternating individual visits were provided by family physicians (FP) within our academic family health team (FHT) in 2016 (see Fig. 1 for description of group perinatal pathway).

This program cares for groups of 8 to 10 pregnant patients and their partners aggregated by month of estimated date of birth (EDB). Each group is co-facilitated by a registered midwife who is embedded within the MSAFHT and two family medicine residents (FMRs). Sessions run approximately two hours and include a short individual clinical exam followed by group discussion of topics related to their stage of pregnancy. The clinical exam includes components of self-care including blood pressure, weight check, pertinent urine testing and vaginal swabs (facilitated by the midwife) and the “belly check” (performed by the FMRs), which is a truncated routine prenatal visit including abdominal palpation and auscultation of the fetal heart. Lab results can be reviewed and necessary requisitions are provided. Patients are encouraged to save non-confidential questions for the group – i.e. postdates management - in order to facilitate discussion, connection and normalization amongst participants. If a longer assessment is required, an individual appointment can be booked. The midwife supervises both residents during clinical visits and facilitates consultation and follow-up as needed. The group discussions are co-facilitated by the midwife and the family medicine residents. There are seven sessions between approximately 16 to 36 weeks gestation and groups reconvene at 6 weeks and 4 months postpartum. A perinatal nurse functions in a coordinating role and other members of the interprofessional MSAFHT join specific sessions as indicated. Alternating with their GPPC sessions, patients also have individual clinical appointments with the staff family physician responsible for their ongoing prenatal and intrapartum care. In this model, approximately half (~ 7) of the prenatal visits occur in the group and the other half are individual visits held between groups. After delivery, the patient’s midwife makes a home visit within 24 to 72 h of hospital discharge to perform the initial well baby exam, support breastfeeding and assess the new parent’s physical and emotional wellbeing. Postpartum group sessions are midwife-led and held at 6 weeks and 4 months postpartum. The postpartum groups are not clinical visits and typically involve discussion about transition to parenthood, mood, infant sleep and feeding as well as an opportunity to debrief birth experiences and socialize with other families. The final 4 to 6 month group session is co-facilitated by the team dietician and focuses on introduction to solid food, with the midwife addressing other questions as needed. Unlike most Centering Parenting groups, these sessions are not individual well baby health assessments. The babies’ medical care subsequently reverts back to their primary care provider after the initial MW home visit.

## Methods

### Data collection

Participants were patients who had participated in GPPC at the Mount Sinai Academic FHT and delivered between November 7, 2016 and October 26, 2018. They were selected from those who indicated interest in providing more extensive feedback when surveyed about the program in 2020. Thirty-eight respondents indicated that we could approach them about the qualitative component. Between July 30, 2021 and May 4, 2022, we invited 20 women to share their experiences. Due to COVID-19 restrictions, we shifted data collection activities from our initially planned in-person focus groups to virtual individual interviews. Two out of 20 individuals declined participation citing personal reasons or recollection concerns and 18 women followed-through with one-on-one telephone interviews lasting between 25 and 60 min. Sessions were conducted by an experienced qualitative researcher who was not a clinical team member and was outside the women’s circle of care, thus mitigating social desirability bias. Participants were assured of confidentiality prior to being interviewed and informed that responses would not impact future care. Only anonymous feedback was shared amongst team members. Interviews were conducted using a semi-structured interview guide developed from literature and also based on the experiences of the research team. The interviewer solicited participant feedback on their experiences with GPC, their perceptions of healthcare providers who they had interacted with during their prenatal and postnatal care, in addition to exploring their recommendations for program changes. We iteratively adapted the interview guide to further probe areas emerging from participant feedback.

All interviews were audio-recorded, transcribed verbatim and de-identified. Participants received a $50 gift card honorarium in appreciation of their time.

Approval for the study was obtained from the Research Ethics Board, Mount Sinai Hospital.

### Data analysis

Six team members (AB, MF, SW, NM, NT, TM) performed line-by-line open coding of the first 6 interview transcripts using an inductive approach without a predetermined theoretical framework. We then met and used principles of the constant comparative method [20–22] to reach an agreement of the codes and create a coding guide. TM then coded the remaining transcripts using the coding guide and the study team reviewed coded data and the supporting verbatim quotes at regular intervals. Themes were subsequently identified from the coded data and informational saturation [23] was reached during analysis of data. We then examined the presence of convergence and discordant responses with disagreements resolved through discussion. To ensure rigor and transparency in the interpretation of results, we maintained an audit trail via memos and meeting notes which documented all major analytic decisions made [24]. We used NVivo (QSR International, Version 12) software to store transcripts and codes and facilitate data management in the study.

Recognizing reflexivity is a way to highlight and value subjectivity in qualitative research as well as an opportunity for authors to examine our positionality and perspectives as we make meaning of our data [25, 26], we purposefully built a research team that reflected the multidisciplinary nature of the work, and that would bring a variety of perspectives, expertise and personal backgrounds to both the research design and analysis. To speak more specifically to our personal and contextual reflexivity: AB, NM SW and MF are all family physicians who provide intrapartum care and have patients within the GPPC model. We range from 10 to 40 + years in clinical practice. We also bring experience from a variety of geographic settings both within and outside of Canada (including the US and the UK). In addition, we are all educators in the Dept. of Family Medicine and have all led and participated in qualitative research related to the care of women and pregnant patients. SM is a registered midwife with both bedside and clinical teaching experience. NT has worked in perinatal care as a RN, MW and lactation consultant. Given that AB, NM, SK, MF, SM and NT all participated in the development of the program (an initiative led by the PI), and have all potentially cared for the participants in our study, we made a decision to add a seasoned research assistant (TM) to our team to conduct our interviews. She brings the perspective of a qualitative researcher removed from our participants’ clinical care.

## Results

Demographics of participants are presented in Table 1. Analysis of the 18 interviews revealed four dominant themes listed and described below. Table 2 provides greater detail of these main themes and the subthemes.

**Table 1:** Participant demographics (*N* = 18)

**Participant Characteristics**	**Interview Participants N (%)**
**Gender**	
• Female	18 (100%)
**Age**	
• Range	29–39 years
• Average	34.24 years
**Marital status**	
• Married	15 (83.3%)
• Common-law/Living with a partner	3 (16.7%)
**First baby**	18 (100%)
**Ethnicity**	
• White	9 (50%)
• Black	1 (5.5%)
• East Asian	4 (22.2%)
• South Asian	2 (11.1%)
• Latin American	1 (5.5%)
• Mixed Heritage	1 (5.5%)
**Highest education level**	
• Completed college or university (undergraduate degree)	7 (38.9%)
• Graduate or professional training (graduate degree	11 (61.1%)
**Combined household income**	
• < $50,000	1 (5.5%)
• $50,000—$100,000	3 (16.7%)
• > $100,000	13 (72.2%)
• Prefer not to answer	1 (5.5%)
**Difficulty making ends meet at the end of the month**	
• Yes	1 (5.5%)
• No	15 (83.3%)
• Prefer not to answer	2 (11.1%)
**Country of birth**	
• Canada	11 (61.1%)
• Other	7 (38.9%)
- Hong Kong	
- India	
- Mauritius	
- Mexico	
- Philippines	
- Poland	
- USA	
**Languages spoke at home**	
• English	11 (61.1%)
• English, French	2 (11.1%)
• English, Other	5 (27.8%)
**Primary care provider (participant)**	
• Mount Sinai Academic Family Health Team doctor	9 (50%)
• Other family doctor	9 (50%)
**Baby healthcare provider**	
• Mount Sinai Academic Family Health Team family doctor or resident	9 (50%)
• Other family doctor	5 (27.8%)
• Other (not specified)	1 (5.5%)
• Pediatrician	3 (16.7%)

1. Participants highly valued information they received from multiple trusted sources.
2. Participants felt well cared for by the collaborative and coordinated interprofessional team.
3. The design of GPPC enabled a shared experience, allowing for increased support of the pregnant person.
4. GPPC facilitated a supportive transition into the community which positively impacted participants’ emotional well-being.

**Table 2:** Main themes & subthemes

**Theme**	**Subthemes**
Participants highly valued information they received from multiple trusted sources	◦ Information was trusted because it came from clinical team members versus external sources ◦ Midwives were particularly valued for their knowledge and support ◦ Informational support eased anxiety and enabled participants to feel better prepared for birth
Participants felt well cared for by the collaborative and coordinated interprofessional team	◦ Patients valued high quality comprehensive care provided by the staff and resident family physicians, midwives prenatal nurse and dietician, all connected to a tertiary care hospital ◦ Women felt that they mattered ◦ Participants felt safe because of effective and consistent interprofessional team communication
The design of GPPC enabled a shared experience, allowing for increased support of the pregnant person	◦ Receiving education with a partner fostered a greater shared pregnancy experience for women ◦ Interactive group learning provided a space for enhanced knowledge acquisition and support amongst first time parents ◦ Sharing experiences mitigated feelings of being alone and enhanced a sense of community
GPPC facilitated a supportive transition into the community which positively impacted participants’ emotional well-being	◦ Participants highly valued how the home visit and group sessions allowed for on-going support postpartum ◦ Relationships built through GPC contributed to mutual support and improved mental health

In general, participants felt that the ensemble of information and support they received while in GPPC allowed them to feel valued, empowered and supported. Furthermore, the connections and the impact of these connections facilitated by the GPPC design extended well beyond the boundaries of the formal program.

1. Participants highly valued information they received from multiple trusted sources

Nearly all participants described the value of the information shared in GPPC sessions. They were grateful to have access to a trusted source who could synthesize what was often described as overwhelming quantities of information into concise and manageable messages. They appreciated that the variety of expertise and lived experience that the GPPC team provided created a richness of information – which was, above all, *trusted*. There was a sense that all information provided had been reviewed and approved by the MSAFHT and was, therefore, credible.

“I liked it…a lot because, when the dietician came to talk to us, I trusted the dietician. That the family program has vetted the dietitian…. they agreed with her view of things… So it felt it was very cohesive care.” (078)

Participants compared this experience to other available sources of information including talking to parents and friends who might have outdated or incorrect information or searching on the internet where they could not ascertain credibility of the content.

“…..having all of the information and being able to actually talk it through with someone, rather than just reading it in a book or on your App. All the way through the pregnancy I felt – I just felt very reassured. I knew that I was getting all of the information I needed to get, to do everything I needed to do, to make sure it was a good pregnancy.” (512)

Particular appreciation was expressed for the midwives’ teaching and care. They were seen as professional, trustworthy, empathic and caring. Participants appreciated their considerable knowledge and the time that they would spend with the group, teaching, fielding questions and supporting individuals. This was considered to be invaluable and central to the GPPC experience. They described how the knowledge that they obtained helped them to manage expectations and prepare for the journey ahead.

“So that I felt like when I went to the hospital, I had a birth plan that was informed by evidence and knowledge, but also my comfort level and what I wanted from it. And I think had I not had that,… it would have been a very different experience, and probably a lot more anxiety.” (543)

They expressed how the information was empowering and also enabled them to advocate more successfully for themselves.

“ …what I didn't know before and then probably wouldn't have thought about as much, was my own recovery and the bonding time and how to kind of protect that and set boundaries with visitors and family. So I think it was just really helpful to have permission to embrace the recovery period. And yeah, how to set boundaries. So that was super helpful, and probably something I wouldn't have thought about or wouldn't have prioritized?” (112)

In essence, participants were describing how receiving this synthesized or curated information provided them with informational support. This support eased the anxiety of their pregnancies and helped them feel better prepared and empowered for the birth experience and beyond.

2. Participants felt well cared for by the collaborative and coordinated interprofessional team

Participants described the value of receiving interprofessional expertise and comprehensive, credible care within the family health team. They appreciated having a holistic support team where midwives, family doctors, resident doctors and other health care professionals worked together in a coordinated way. Participants valued the combining of their medical care with perinatal education. They consistently felt supported in this environment and valued the contribution of each member of the team. They appreciated the family medicine “medical home” as being more of a community and less transactional than their speculation as to what they might have received during routine perinatal care. They trusted their team of health professionals and felt that they were in good hands.

“…it felt very co-ordinated and it felt like it all came together so I didn’t feel like the prenatal classes were independent of my relationship with my family physician or with the prenatal nurse. It felt like I was getting excellent care that was, that covered everything that I needed from different people who were in different parts of the family medicine group that were co-ordinated in working together”.(001)

Participants described trusting relationships with the family physician and appreciated the continuity of care provided by the team of clinicians who offer prenatal, intrapartum, postpartum and newborn care.

Participants appreciated the roles that all the other members (including midwives, dietician, GPPC nurse coordinator) of the interprofessional team played in their care as well as the fact that the team communicated and coordinated clinical information effectively.

…. And because there’s that continuity in terms of they had access to my records. They knew what was happening. “(547)

We asked participants about their perception of the role of the family medicine resident in their perinatal care. Participants’ feedback was that they understood they were receiving care in a teaching setting and, in general, were open to resident participation in their perinatal education and clinical care. They understood all resident care was supervised and that the residents would consult the staff physician or midwife when they had questions. Residents were viewed as engaged in the GPPC process and attentive to patient care. In fact, at times the residents were seen as more open and attentive than the staff physicians.

“..I felt like sometimes my resident had a little more time to listen than the physician did. And all the care she provided was excellent so I had a lot of confidence in her and yeah, I was very appreciative of her role in my care as well…” (014)

However, some participants expressed some hesitation about the residents’ knowledge and clinical maturity.

"I feel like the residents were not experienced enough to...they had no clue about L & D or newborns, everything was based on textbooks, which I think all of us kind of felt they just weren’t able to answer any questions that we had..” (509)

Our participants highlighted the support they received from their “village” of GPPC professionals at different times in the pregnancy and birth journey. They trusted their team and felt enveloped and safe in their care.

“ I think it's good to have integrative because I feel like there's – again, it's a community, so strength in numbers and you're able to have it all dealt with, and everybody's on the same page. So I think there's definitely pros to having that… I might have missed something – they might have missed something… I didn't realize at the time how …. it was just a really integrative experience and I had no idea how thorough it was until, you know, looking back on it now” (537)

Participants also acknowledged feeling valued and that they mattered.

“You get everything. I feel like I got pretty much everything from the day one…there's comfort in everything in one place, and the aftercare. I think it's amazing…I guess, it is service but it's also, you feel like you’re a valuable patient, and you’re not just another mom who’s giving birth. (040)

3. The design of GPPC enabled a shared experience, allowing for increased support of the pregnant person

Much of the value of GPPC was attributed to it being a shared experience – with other pregnant individuals going through the same experience in the same time frame and with their partners/support people. The organic development of the group, facilitated and fostered by the GPPC design and process, was critical to their experience. The structure of GPPC is intentional in that it groups pregnant people by EDB and includes partners or other support people. Almost all of the respondents commented on the value of sharing this experience with others at a similar stage in their pregnancy.

“And so that was just an absolutely phenomenal support system to have those other moms to share the experience with and go through it at the exact same time………if it was not for this group being in exactly the same stage at the same time, I can conclusively say my mental health would definitely have been worse.” (512)

They felt comfortable in the group setting and with the interactive learning. They described the value of hearing about other’s experiences and their questions and concerns. They valued the support that they received from each other at different stages of the journey and appreciated being part of a collective (and benefitting from the collective wisdom of the group.)

“..you potentially get to meet others and people are asking questions you haven’t thought of……. And also, a sense of affiliation. “(088)

For women that were isolated from family or did not have a network of personal supports, the group served as the village they did not have. Sharing the perinatal experience with other participants allowed them to feel less alone. In fact, many of those participants expressed that they “wouldn’t have known what to do” without the program.

“I did not have a group of friends – I didn’t have anybody close to me – living here in the same city…So a lot of that element of the emotional support, I honestly just think I would not have gotten it. It just wouldn’t have been there…I think that’s an impossible benefit to really recreate – the Group Care.” (512)

Participants described the importance of including their partner. This inclusion meant that the partners heard the same information and thus were able to support them better during pregnancy and after the birth. It also helped the partners to feel that they were an integral part of the process as they were often able to ask questions that the mother to be had not thought of.

“…we were armed with the same information so when we had to make a decision or when there was something challenging, one of us could say ‘oh I remember in the class we did this or that’, we could make sense of it and talk about it, get a shared base of knowledge. (001)

And, finally, respondents expressed how their partners found it helpful to connect with other partners and support people in this pregnancy and birth journey.

“But I think going through the process it really helped him I think talking to other dads, dads to be……. Again he felt that sense of comradery, and support. So you know for him like it was very, very useful.” (547)

4. GPPC facilitated a supportive transition into the community which positively impacted participants’ emotional well-being

Although the bulk of the formal GPPC process was experienced in the clinic setting, the program had a much broader impact. The participants reflected on how their experience of the labour, birth and hospital postpartum process had been affected by the group process. And that impact continued as they transitioned from hospital, to their home and into their community.

Participants commented on how challenging the postpartum period can be and how vulnerable they felt as new mothers. They reflected on how the relationships they had built through GPPC provided support in multiple ways and increased their confidence and competence.

“…it gave a lot of reassurance OK, you're not alone. There's the sleepless nights. And there's a light at the end of the tunnel when you have someone also walk through the exact same thing as you. You just feel OK, I can do it. We can do this together type thing. And it actually helped a lot with your mental well-being. I think the first, probably the first three, four months you're kind of going crazy because it's your first baby and your whole life has completely changed just 180. And having someone go through that with you as multiple women go through that with you. It gives you a lot of reassurance.”(090)

The home visit by the midwife was a highlight of the program for many participants. It was valued as an opportunity to debrief about the physical and emotional aspects of the birth, provide breastfeeding support and reassurance about baby’s wellbeing in the comfort of their home environment.

In addition, participants appreciated the instrumental support of breastfeeding teaching in the immediate postpartum period and the reassurance that the home environment was suitable for the new baby.

“That [home visit] was a nice thing for us… my baby was crying literally all night, we were crying all night. It was terrible, she came and I was a mess. So it was life saving for us....”(008)

“..she was able to calm me emotionally and help me physically of how to deal with engorgement and breastfeeding, how to latch and things like that.” (093)

Participants reported how many group members organically started to connect outside the structure of the GPPC sessions and how this connection continued through the postpartum period and throughout the babies’ first year. These strong support networks were particularly important in the postpartum phase where they shared information and encouraged each other through the challenges of early parenthood.

“So we were able to, message each other and things like that. So it really, it helps with the anxiety aspect of it, especially if you're worried. Am I doing this right? Or if you have any tips for breastfeeding, things like that. So it helped in that aspect. And that helped with my mental state, I think, a lot.”(090)

In fact, by the time that we were interviewing these parents, many had had their second child and were still meeting with and feeling supported by the connections that had been made during GPPC.

Woven throughout all of the interviews was the observation that the support structures created by GPPC positively impacted mental health. Participants reflected on the meaning of the group experience and connected their experience to their mental wellbeing.

“I think from a mental health perspective, too, I definitely face a lot of anxiety……. And being in that environment, that group environment, really helped with the perinatal mental health aspect of it. (547)

Relationships that participants formed with multiple healthcare professionals – family physician, resident, midwife—and other GPPC participants helped with feelings of isolation and loneliness. Participants reported feeling well-supported with consequently better mental health throughout pregnancy and early parenthood.

“It (GPPC program) was absolutely vital to my mental health……….the group provided that supplemental support and another avenue for me to ask questions. Or share my emotions and anxieties about what was happening.” (512)

Thus, GPPC, which started during the early stages of participants’ pregnancies, fostered the building of a community which stretched far beyond the physical confines of the space in which most of the program took place. This community, which existed over time and in different settings, had a positive impact on participants’ mental wellbeing and adjustment to parenthood.

“I truly believe that when it comes to raising a child, it really does take a village. And what I mean by that is not that you should let other people raise your child,… it’s a community, right? You need to sometimes lean onto other people to see what they’re doing and to get insight into how to prepare or what to expect or learn from their experiences.” (537)

Participant feedback about GPPC was overwhelmingly positive with no dissenting views noted. However, when prompted for gaps or feedback, participants came up with some recommendations for future program structure. These were not consistent across all interviews and included: 1) separate sessions for women and partners, particularly for issues considered sensitive by women; 2) smaller breakout groups to encourage greater participation from those not comfortable in large groups; 3) more teaching and resources about breastfeeding and the use of infant formula; 4) additional home visits postpartum.

## Discussion

“It takes a village to raise a child.” This African proverb came to our collective mind as we began to review the interview transcripts and our impression was reinforced when a participant used the quote in one of their responses. It became clear very quickly in our data analysis that social support was central to the value that participants found in GPPC. Recently published research by Renbarger et al. supports this interpretation [11].

Support is the common thread within our main themes. Reflecting on their GPPC experience, our participants highlighted all four constructs of social support described by Renbarger which built on previous work done by House and Heaney [27, 28]. Participants described receiving informational support from their healthcare providers and peers. This knowledge was critical to their pregnancy and birth journey, helping them feel prepared, adjusting their expectations and allowing them to engage with the rest of the health care system in a knowledgeable way. Participants also described the importance of the emotional and appraisal support that they received from their peers, partners and healthcare providers. This mitigated their anxiety and empowered them to advocate for themselves. The participation of their partners in the process and the support of the whole group made them feel less alone during challenging times. The midwife, in particular, was seen as a source of all four types of social support. In addition to providing informational and emotional support, she was seen as providing tangible and appraisal support in the form of breastfeeding coaching and postpartum care in the home.

These findings are very consistent with other qualitative studies of GPC. Herrman’s study of five focus groups of GPC participants describes four themes: 1.It’s about respect 2.Knowledge is power 3. I am a better mother and 4.Supporting each other [10]. Although we did not use the word “respect” in our findings, our participants felt valued and the importance of communication and trust (descriptors in Herrman’s theme of respect) were also clear in our data. Herrman’s other substantive themes are echoed within our findings. Hunter found that Pregnancy Circles functioned as “an instrument of empowerment, mediated through increased learning and knowledge sharing, active participation in care and peer and professional relationship building” [29]. Similarly, our participants described how learning from trusted sources gave them the basis to know what to expect going forward and to advocate for themselves.

The impact of social support on mental health was expressed repeatedly by our participants and is clearly woven into each of our four themes. Improvements in mental health as a result of GPPC has been documented in the literature. A meta-analysis demonstrated improvement in psychological outcomes for adolescents and low income women participating in group prenatal care [30]. In a qualitative study comparing group prenatal care with traditional care, group participants experienced mental health benefits through the education and preparation function of prenatal care and the supportive group environment. These benefits included reducing stress, increasing knowledge and motivation, and supporting health care engagement [31]. Interestingly, our participants seemed to express the impact of their group experience on their mental health more overtly.

The model of GPPC offered at the MSAFHT is unique in that it is co-facilitated by a MW and a FMR. We were not surprised at the unmitigated enthusiasm for the MW’s contribution. Similar sentiments were described in Hunter’s study where participants described “traveling together” with midwives who knew them well and remembered their stories. Given the structure of our unique program, we explicitly sought our patients’ perceptions of the FMR’s. It was clear that participants understood that FMRs are learners and that they might have to check with staff physicians or the midwives in certain circumstances. They described how the residents had more time to address concerns than the staff physicians and in many instances expressed appreciation for their attentiveness. In fact, in some cases, the patients saw the resident as the primary provider for themselves and their baby.

Our unique, hybrid model of group perinatal care and individual care is different from the classic “Centering Pregnancy” programs that have been evaluated in the literature. As we look at the impact of this program on our patients, it is impossible to tease out which parts relate to the group dynamics, to the continuity of care provided in the academic family health team, to the MW home visits or the interprofessional sessions involving MWs, a dietician and FMRs. Although this makes it impossible to fully compare to other studies, our data shows that this approach to perinatal care is possible in an academic FHT and the participants describe significant impact. Most family physicians are unable to participate in “classic” GPC due to time and other competing constraints. However, this model which incorporates other members of the family health team shows that a hybrid model can be built which preserves the key elements that are necessary for success. In addition, it demonstrates that it is possible to do it in the teaching setting by adapting and adding some innovative elements. Resident involvement did not diminish the extremely positive impact on participants while achieving educational goals set by the program. The full integration of midwifery into an Ontario family health team is also a unique aspect of this novel program.

Our participants were interested in receiving more home visits by the midwives. This is not surprising given the support in the literature for home visits which have been described by Dahlberg as providing “relational continuity” [32] and the opportunity to debrief the birth experience during the vulnerable early postnatal period while breastfeeding was being established. Another suggestion by our participants that some sessions might not include partners is also congruent with the literature [29, 33] where some women expressed reservations about including partners and there were concerns that the inclusion of partners would inhibit the formation of relationships between the women [32]. However, it appears that in our study, the benefits of including their partners far outweighed any reservations.

The GPPC team provided the start of the “village”—purposefully built to support to these families as they transitioned from prenatal care to the intrapartum and immediate postpartum period in the hospital. This support continued into their homes and communities as they began their parenthood journey. However, the support from the healthcare team was magnified by the relationships that participants had deepened with their life partners and forged with the peers that they had met on this journey. Thus, the village grew.

We can also look at our results through the lens of social network theory [34]. Participants in GPPC at the Mount Sinai Academic FHT, became part of a social network for their labour, birth and early parenting journey by developing relationships with their healthcare providers, partners and fellow participants through the group process. Although we did not conduct a social network analysis on our data, we can hypothesize how the relationships developed between the participants (mostly in dyads) that arrived at the first GPPC session and the network of health care providers and other pregnant people. The strength and directionality of these relationships would have changed throughout the course of the pregnancy journey and postpartum period. The support of the peer group assumed greater importance as they navigated the uncertainties of the postpartum and newborn period and had fewer formal group sessions. For many participants, these relationships persisted for several years after the program had formally ended. Thus entirely new social networks were created.

We were struck by the maxim that “the whole is greater than the sum of its parts” when considering the impact of GPPC on our participants. The relationships which were forged were multi-faceted and had profound impact on participants’ transition to parenthood. Many felt that the entire experience was their best interaction with the health care system, leaving them feeling valued and empowered as also described by Hunter [29]. Our participants went further in that they alluded to the program filling in for missing relatives and friends to guide them through this momentous experience. In essence, they were missing the “village” of extended family and social structures which have supported new parents in other eras or cultures. These feelings were expressed by our participants who gave birth prior to the COVID-19 pandemic and we know that many aspects of the pandemic have resulted in increasing rates of perinatal mood disorders [35, 36] and increasing needs for support among birthing people [37]. Our GPPC program was converted to a virtual group at the start of the pandemic. The prenatal clinical visits were no longer integrated into the perinatal education and the midwife’s home visit was canceled for the first year. Although we have not surveyed or interviewed those who experienced “COVID era GPPC” our sense is that it was a completely different experience. There was still information sharing, but the relationships did not form as strongly without the opportunity to spend many hours together in the same physical space. Sadly, these are the parents who need even more support due to the fears, social isolation and illnesses of the pandemic.

### Limitations

Our participants were well educated, affluent, predominantly Caucasian and partnered. They were a self-selected group – in that they elected to participate in GPPC, responded to the survey and volunteered to be interviewed. Thus it is difficult to generalize our findings. On the other hand, despite such external markings of privilege, they appeared hungry for the connections and support provided by GPPC.

COVID-19 affected consistency in the timing between GPPC and follow-up interviews; this varied between forty five to fifty six months. Longer participation intervals may have impacted subjective memory for some of the women we interviewed. We could not identify the exact lag time for individual participants due to anonymization of the data.

## Conclusion

Our qualitative findings reveal that the participants’ experiences with the GPPC program can be interpreted through the lens of social support and social networks. We have demonstrated that even in this era of instant access to information and constant electronic connection to others, our participants describe the irreplaceable need for in-person information sharing, social connections and trust to help navigate the challenges of becoming parents. We conclude that it will always “take a village to raise a child” and that we, as health care providers, can play a role in creating that village.

## Data Availability

The datasets obtained during the current study is not publicly available due to confidentiality but may be available from the corresponding author on reasonable request.
